# Current perspectives and trends of neutrophil extracellular traps in organ fibrosis: a bibliometric and visualization study

**DOI:** 10.3389/fimmu.2025.1508909

**Published:** 2025-03-05

**Authors:** Yanbo Li, Zhengmin Cao, Jing Liu, Rui Qiang, Jiuchong Wang, Wenliang Lyu

**Affiliations:** ^1^ Department of Infectious Diseases, Guang’anmen Hospital, China Academy of Traditional Chinese Medicine, Beijing, China; ^2^ Department of Oncology, Beijing Hospital of Traditional Chinese Medicine Shunyi Hospital, Beijing, China

**Keywords:** bibliometrics, visualization, neutrophil extracellular traps, organ fibrosis, neutrophil, immune response

## Abstract

New insights into the role of immune responses in the fibrosis process provide valuable considerations for the treatment of organ fibrotic diseases. Neutrophil extracellular traps (NETs) represent a novel understanding of neutrophil functions, and their involvement in organ fibrotic diseases has garnered widespread attention in recent years. This study aims to conduct a bibliometric analysis and literature review focusing on the mechanisms by which NETs participate in fibrotic diseases. Specifically, we utilized a bibliometric dataset that includes 220 papers published in 139 journals, originating from 425 organizations across 39 countries, with a total citation count of 12,301. Keyword co-occurrence analysis indicates that the research focus on the mechanisms of NETs in organ fibrosis is likely to center on NETosis, immune responses, immune thrombosis, inflammation, and tissue damage associated with NET formation. In conclusion, our findings underscore the current status and emerging trends in NET research related to organ fibrosis, offering novel insights into the mechanisms by which NETs contribute to the pathogenesis of fibrotic diseases, as well as potential therapeutic strategies.

## 1 Introduction

Fibrosis is a disease characterized by the excessive deposition of extracellular matrix and the destruction of normal parenchymal structures, leading to organ dysfunction (1). Fibrosis can affect various organs and systems throughout the body, including the lungs, liver, heart, kidneys, and skin, resulting in a significant disease burden. Epidemiological data indicate that the annual incidence of major fibrosis-related diseases is approximately 4,968 cases per 100,000 population (2). The pathological process of fibrosis involves dynamic interactions among various cell types. When tissue damage occurs, locally released chemokines attract immune cell populations from the circulation to the site of injury, including neutrophils, monocytes, and macrophages. These immune cells produce and release a multitude of pro-inflammatory and pro-fibrotic factors, stimulating the activation and abnormal proliferation of fibroblast (3, 4).

Neutrophils are the most abundant type of effector cells and granulocytes within the immune system (5). Once activated, neutrophils can release NETs through a special way of programmed cell death, which is called NETosis (6). Recent studies have elucidated the intricate mechanisms underlying NETosis (7). The primary structural component of NETs is nuclear DNA, and chromatin condensation is an essential prerequisite for its extrusion into the extracellular space. A key event in NETosis is the condensation of chromatin, a process mediated by PAD4-induced histone citrullination (8). The nuclear membrane serves as the initial physical barrier to chromatin release. The rupture of this membrane is facilitated by phosphorylation events, including PKCα-mediated phosphorylation of lamin B (9) and CDK4/6-mediated phosphorylation of lamin A/C (10). The plasma membrane represents the second physical barrier to NET release. Its rupture is primarily associated with the disintegration of the cortical cytoskeleton, including the degradation of actin filaments, microtubules, and peripheral vimentin cytoskeleton (11). Additionally, the actin cytoskeleton is regulated by Rho kinase, which plays a role in the early nuclear translocation of PKCα and CDK4/ (12).

Recent studies have highlighted a significant link between NETs and various organ fibroses, including pulmonary and myocardial fibrosis. NETs contribute to tissue damage through mechanisms such as immune thrombosis, sterile inflammation, and immune dysregulation, driving the progression of fibrotic diseases (13, 14). However, a comprehensive, evidence-based analysis focusing on the current state of research and future directions in the context of NETs and fibrosis remains lacking. This study aims to fill this gap by providing an overview of the latest research trends. Using bibliometric and visual analysis of Web of Science (WOS) publications from 2010 to 2023, we identify key research areas, including NETosis, NET-mediated immune cell crosstalk, NET-induced fibroblast activation and cytotoxicity, and NET-driven immune thrombosis. By synthesizing existing knowledge, this study offers a comprehensive, evidence-based resource for advancing research in the field of NETs and fibrotic diseases.

## 2 Method

### 2.1 Data search and retrieval strategy

Literature from Web of Science Core Collection database between January 1, 2010 to September 31, 2023 was downloaded for this study. See search strategy in the Supplementary Information. To ensure accurate interpretation of the results, only article and review in English were included.

### 2.2 Data process and visualization

To visualize collaborations between countries, institutions, and authors, as well as to analyze co-citations and keyword co-occurrences, VOSviewer version 1.6.16 was used. A burst detection analysis of keywords was conducted using CiteSpace version 6.2.R6. In addition, Scimago Graphica provided visualization support for the analysis. To eliminate redundant entries, synonymous expressions were manually standardized. For instance, “liver fibrosis” and “hepatic fibrosis” were merged.

## 3 Result

### 3.1 Annual publications and citation

**Figure 1:**
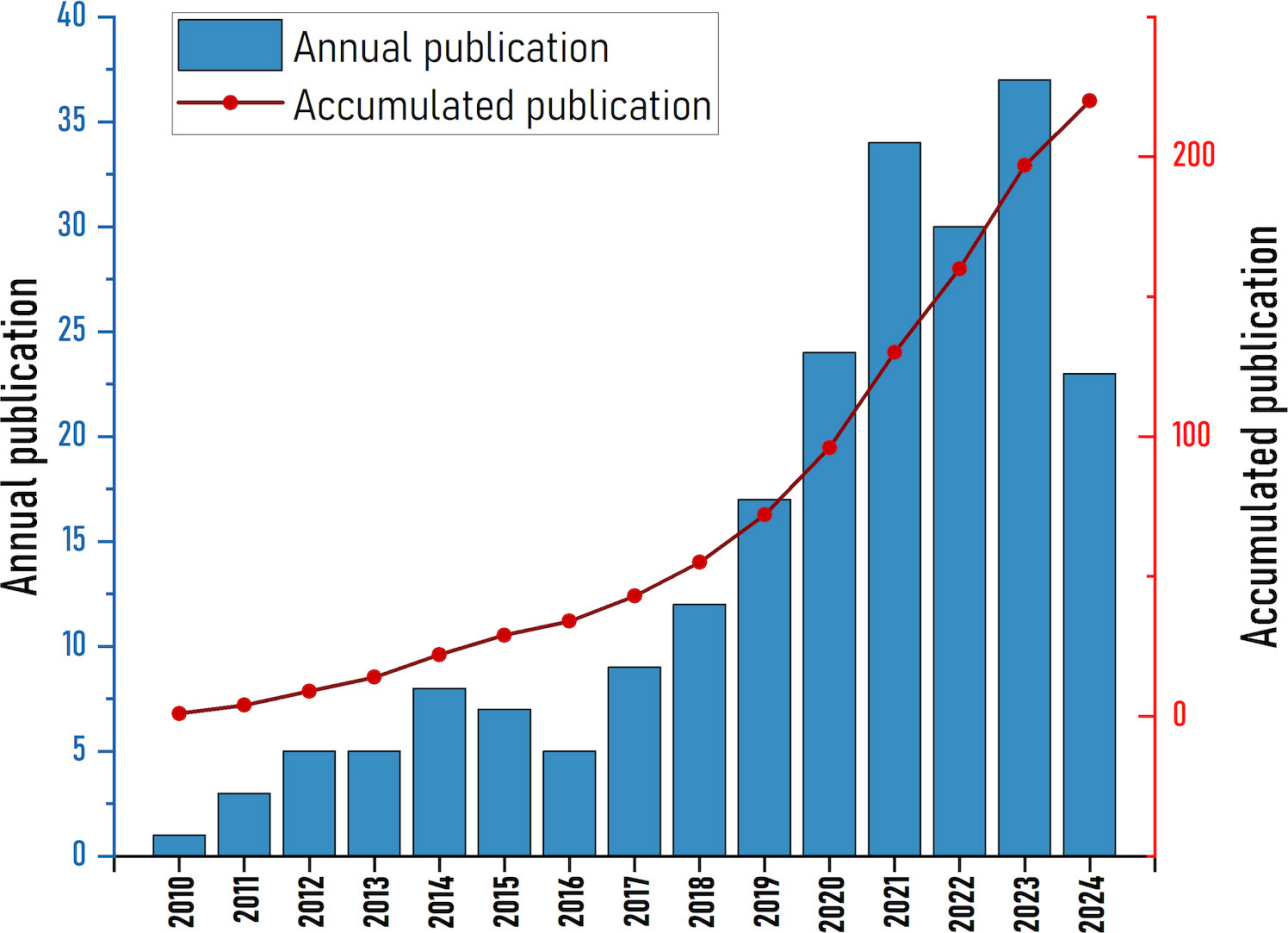
Global publication output on NETs in fibrosis diseases from 2010 to 2024.

The study contains 220 papers from 39 countries, 425 organizations, published in 139 journals, and 12301 quotes. **Figure 1** shows the annual number of publications and citation frequency for the period 2010 to 2024. In general, the number of annual publications has increased steadily and quickly. The involvement of NETs in the etiology of fibrotic disorders was not studied before 2010. The number of articles increased slowly between 2010 and 2018, and the research was still in its infancy. The rate of publications started to pick up speed after 2018. In addition, omitting data for 2024 due to incomplete data, there is significant increase in annual postings to over 30 in 2021, 2022, 2023. We hypothesize that this may be related to the increased heat in the NETs research field (15).

### 3.2 Distribution of countries/regions

**Figure 2:**
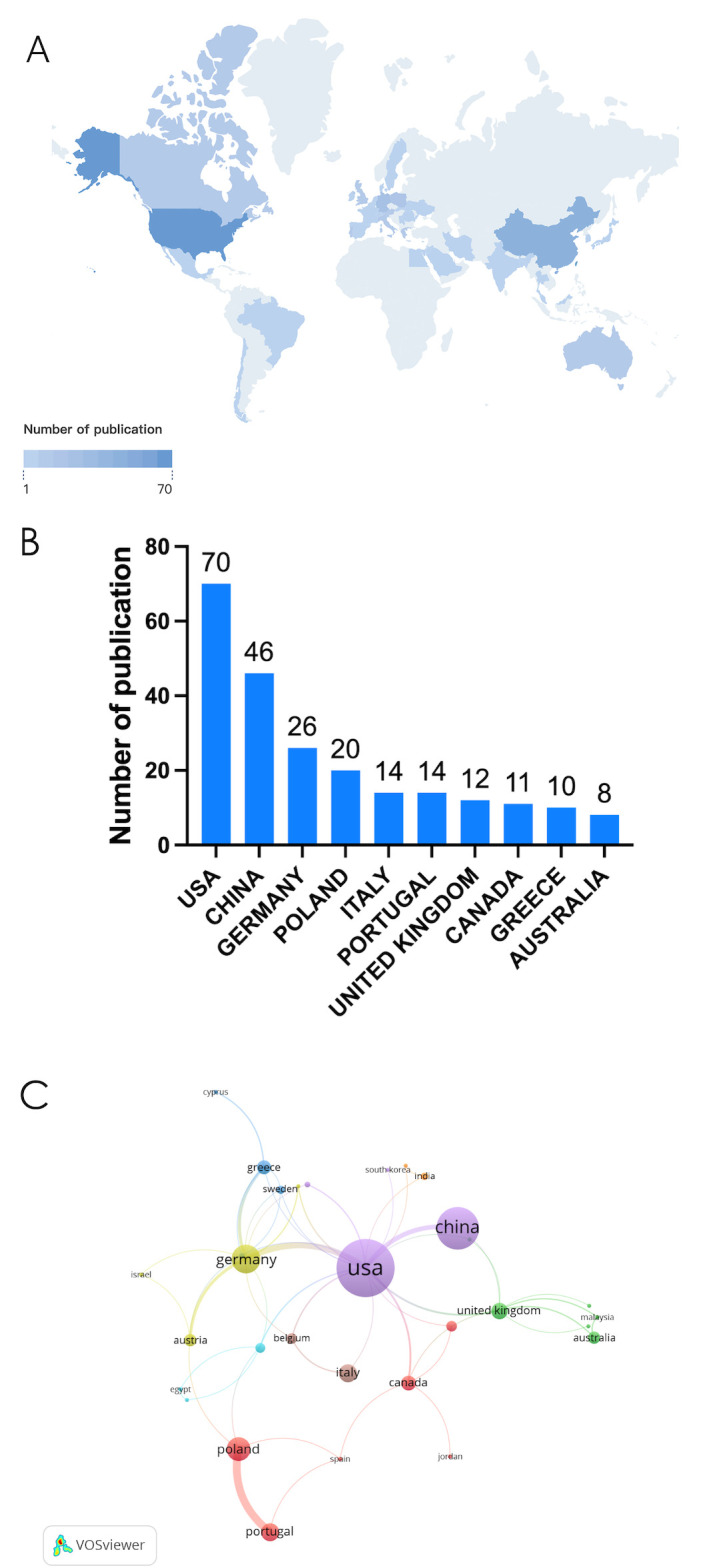
**(A)** Geographic distribution map based on the total volume of publications for different countries/areas. **(B)** The top ten countries/regions in total number of publications. **(C)** Visualization map of country/region citation networks generated using the VOS browser. The thickness of the lines reflects the strength of the citations.

Currently, a total of 39 different countries/regions have published studies on NETs in relation to fibrotic diseases, as shown in **Figure 2**. Papers in related fields have been published mainly in North American, Asian, and European countries. **Figure 2B** shows the number of publications in these countries/regions. The United States had the most publications (31.81%, 70), followed by China (20.90%, 46) and Germany (11.82%, 26). **Table 1** shows the top 10 countries/regions. **Figure 2C** shows that in our analysis of global collaborations using the VOS observer, we found that the main links between countries/regions were concentrated between North America and Europe and America, and between North America and East Asia.

**Table 1:** Top 10 countries / regions with the highest number of publications.

rank	country	documents	citations	total link strength
1	USA	70	3033	42
2	CHINA	46	898	9
3	GERMANY	26	1572	30
4	POLAND	20	380	17
5	ITALY	14	260	3
6	PORTUGAL	14	165	15
7	UNITED KINGDOM	12	474	12
8	CANADA	11	860	7
9	GREECE	10	712	10
10	AUSTRALIA	8	239	4

### 3.3 Contributions of institutions

**Figure 3:**
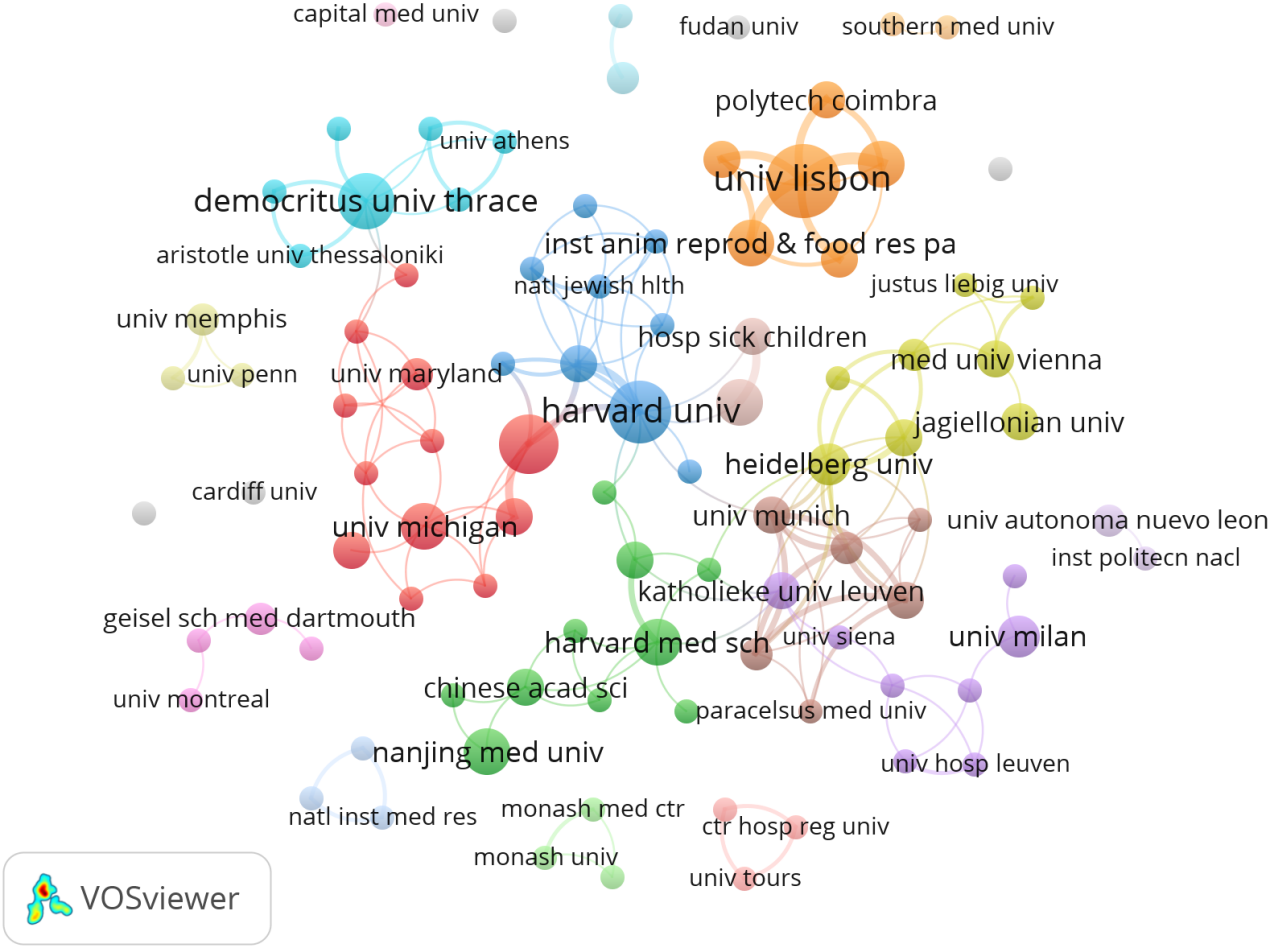
Visualization of institutional collaboration networks.

CiteSpace generates a network visualization map of institutional collaboration. The paper on NETs in the study of fibrotic diseases includes contributions from 425 institutions. The United States and China have a large number of institutions engaged in scientific research. Most of the articles were published by the University of Lisbon, Harvard University, University of Georgia and Democritus University of Thrace from the United States, Sweden and Greece. The VOS viewer presents inter-agency collaboration, as shown in **Figure 3**. It is found that the University of Lisbon has the highest total connection, but its cooperation with other institutions is limited; Chinese scientific research institutions, represented by Nanjing Medical University and Chinese Academy of Science, and Harvard Medical School formed a green group, suggesting wider cooperation and exchanges.

### 3.4 Journals and co-cited journals

A total of 139 journals have published articles in the field of NETs and fibrotic diseases. **Table 2** lists the 10 journals with the largest number of publications. The journal with the most published papers is Frontiers in Immunology (N=17), followed by International Journal of Molecular Sciences (N=10) and Plos One (N=6). The top three cited journals are Frontiers in Immunology (N=569), Journal of Immunology (N=480) and Plos One (N=427). Among the top 10 magazines, there are 3 journals in JCR Q1 and 7 journals in JCR Q2, and the IF value of 8 journals exceeds 5 points, among which the journal with the highest IF value is Autoimmunity Reviews (IF=8. 3).

**Table 2:** Top 10 journals with the highest number of publications.

Rank	Journal	Count	IF	JCR
1	Frontiers in Immunology	17	5.7	Q1
2	International Journal of Molecular Sciences	10	5.6	Q2
3	Plos One	6	3.7	Q2
4	Journal of Clinical Medicine	5	4.6	Q2
5	American Journal of Respiratory Cell and Molecular Biology	4	5.8	Q2
6	Autoimmunity Reviews	4	8.3	Q1
7	Biomedicines	4	5.2	Q2
8	Biomolecules	4	5.2	Q2
9	Frontiers in Pharmacology	4	5.4	Q2
10	JCI Insight	4	8.6	Q1

**Table 3**. Top 10 journals in terms of citations. Frontiers in Immunology (569 co-citations) was the most cited magazine, followed by Journal of Immunology (480 co-citations) and Plos One (427 co-citations). Among the top 10 journals, the journal with the highest impact factor is Nature Medicine (IF=89. 8), followed by Science (IF=83. 4) and Blood (IF=22. 8).

**Table 3:** Top 10 most cited journals.

Rank	Co-cited journal	Citations	IF	JCR
1	Frontiers in Immunology	569	5.7	Q1
2	Journal of Immunology	480	4.9	Q2
3	Plos One	427	3.9	Q2
4	Blood	418	22.8	Q1
5	Nature Medicine	328	89.8	Q1
6	Science	295	83.4	Q1
7	American Journal Of Respiratory And Critical Care Medicine	284	20	Q1
8	PNAS	280	11.2	Q1
9	Journal of Clinical Investigation	258	13.3	Q1
10	Journal of Experimental Medicine	225	13.9	Q1

**Figure 4:**
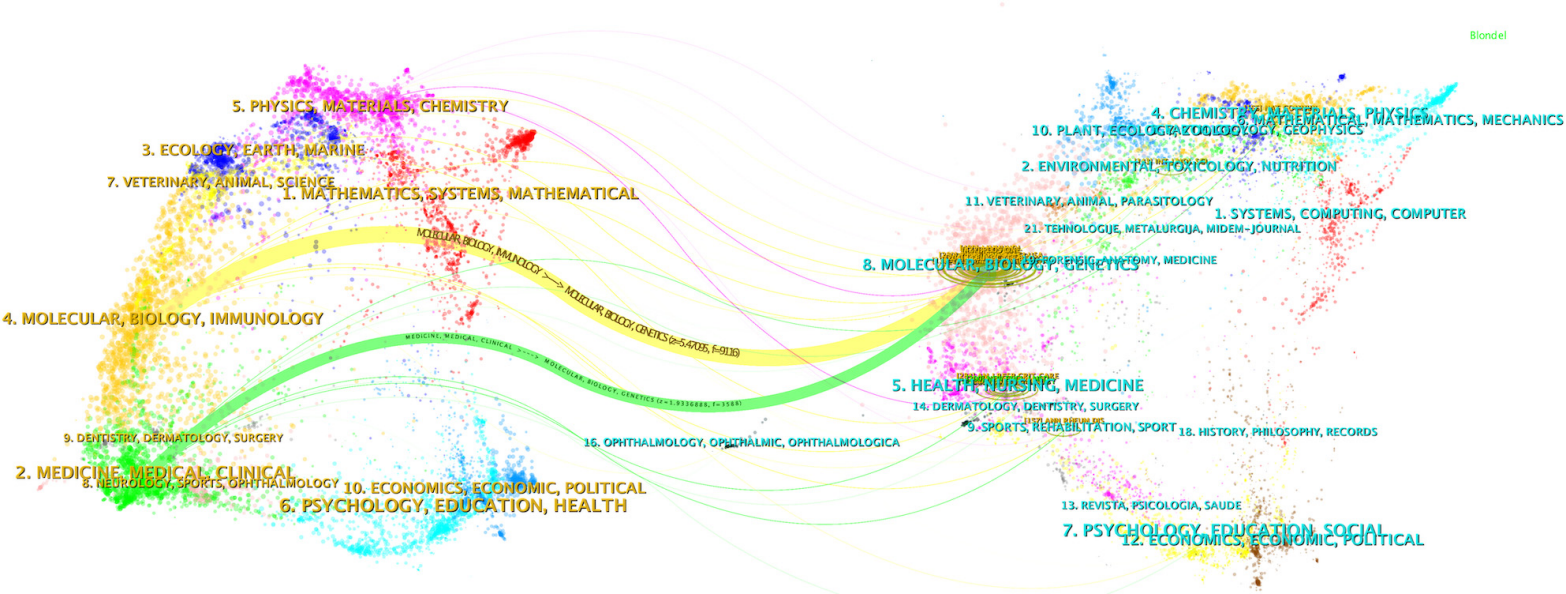
Dual-map overlay illustrating the journals where the research was published (left) and those that cited it (right).

**Figure 4** shows dual-map overlay of journals in which research was published. The cited journals were displayed on the right side of the map, and the citing journals were on the left. Reference paths are shown in different colors. The width of the pathway is related to the frequency of being cited. Currently there are 2 main citation pathways, which means that articles published in Biology/Molecular/Genetics journals are usually cited by studies published in Molecular/Biology/Immunology and Medicine/Medical/Clinical journals.

### 3.5 Authors

**Figure 5:**
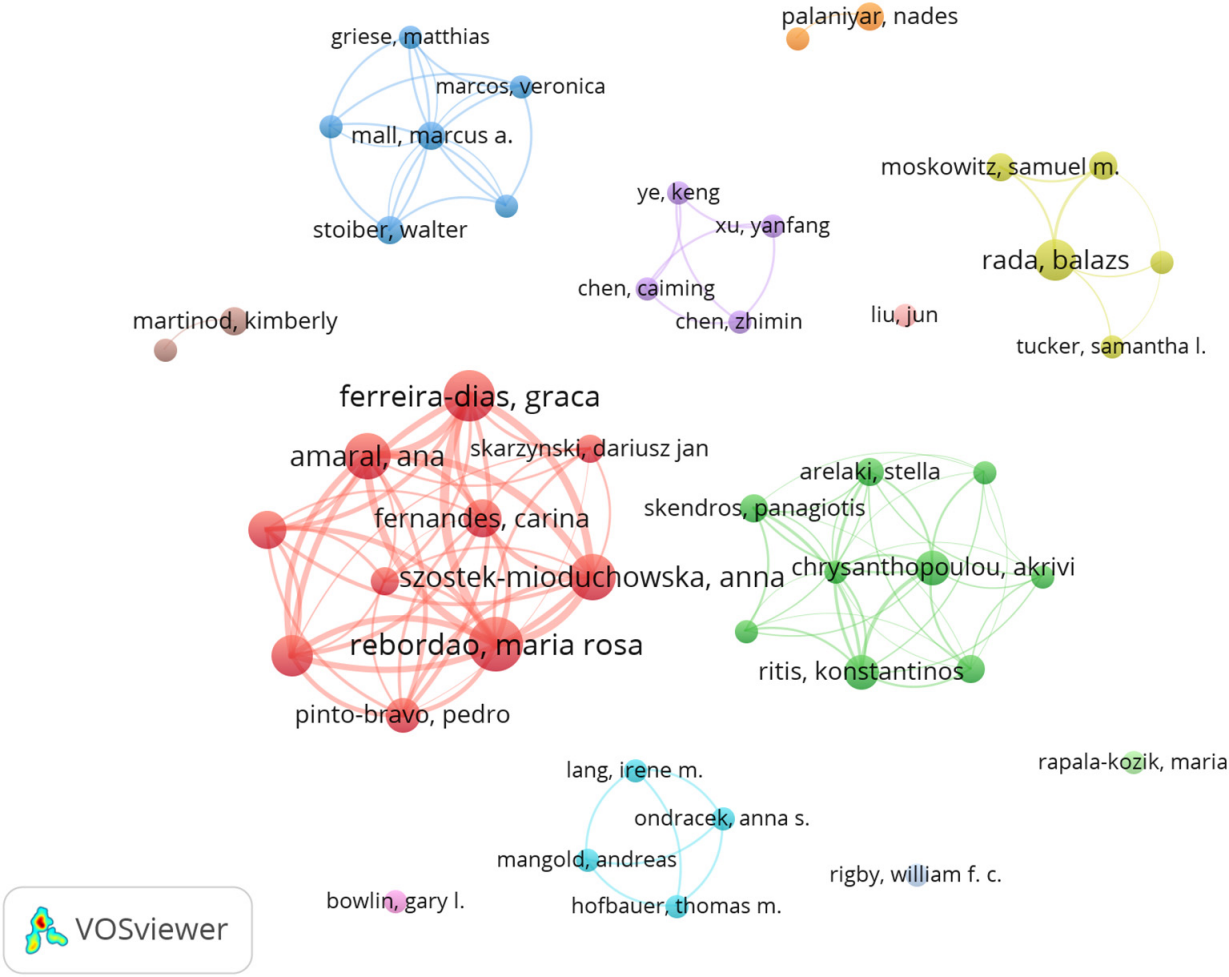
Authors’ collaboration network of research on NETs in fibrotic diseases.

A total of 1574 authors were identified as contributors to the field during the literature search. **Table 4** summarizes the top 10 authors by the number of publications. Rebordao Maria Rosa and Ferreira-dias Graca have the most publications, with 13 and 12 papers respectfully, indicating their leading achievements in the field. The author cooperation network diagram constructed by VOSviewer shows that there is a strong cooperation relationship between high-yield authors and research teams in the field (**Figure 5**). The thicker the line between the authors, the more they have published together. Rebordao Maria Rosa and Ferreira-dias Graca are leading researchers in this field, and their cooperation network is the most extensive and influential.

**Table 4:** Authors in the top 10 publications.

Rank	Authors	Count
1	Rebordao Maria Rosa	13
2	Ferreira-dias Graca	12
3	Amaral Ana	10
4	Szostek-mioduchowska Anna	10
5	Lukasik Karolina	8
6	Rada Balazs	8
7	Fernandes Carina	7
8	Skarzynski Dariusz J.	7
9	Chrysanthopoulou Akrivi	6
10	Pinto-bravo Pedro	6

### 3.6 References with citation bursts

**Figure 6:**
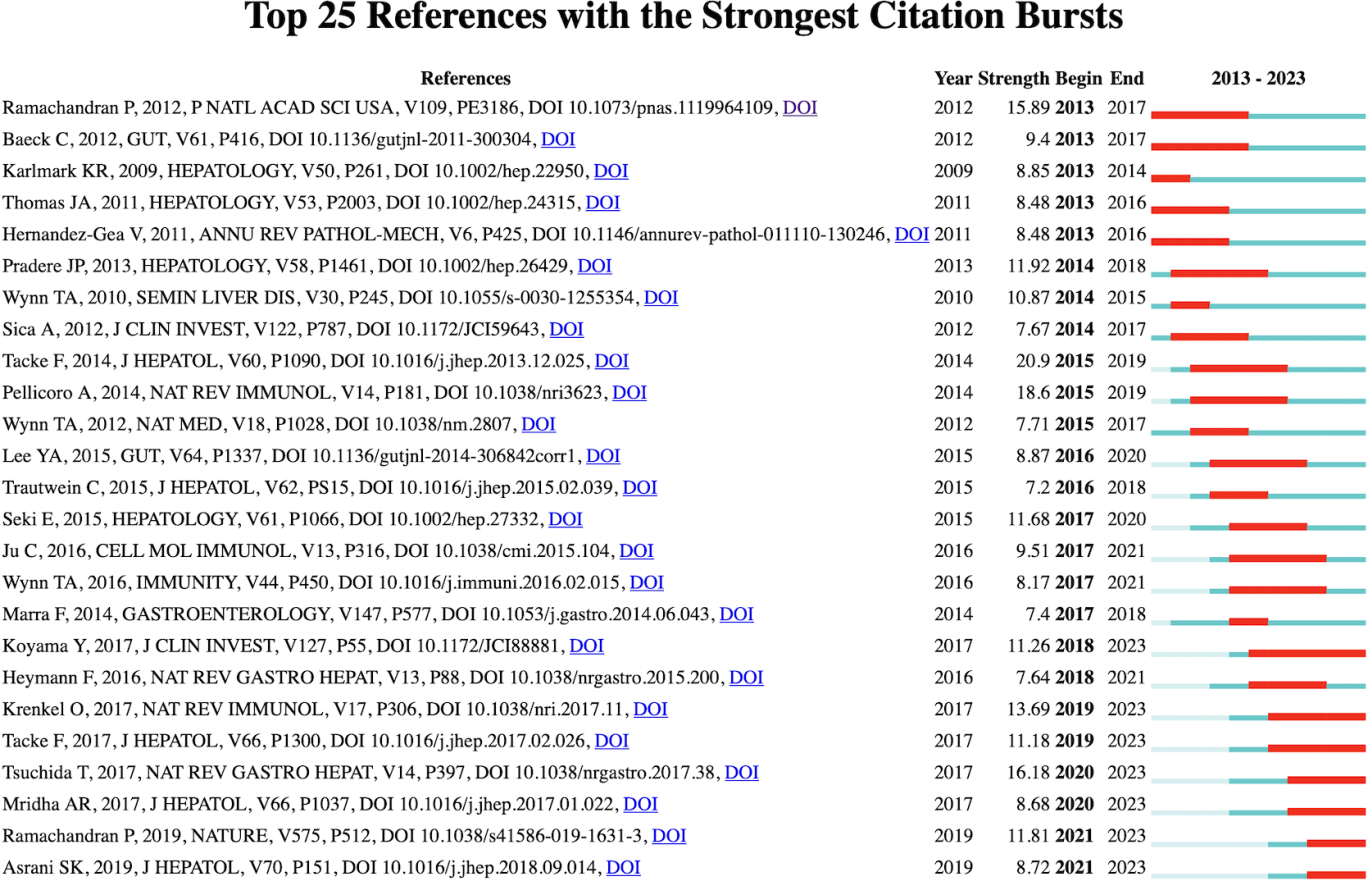
Visualization map of top 25 references with the strongest citation bursts from 2007 to 2020.

**Figure 6** shows the 25 most frequently cited documents. The earliest citation burst occurred in 2007, and the latest in 2021. Among these references, “ Cystic Fibrosis Sputum DNA Has NETosis Characteristics and Neutrophil Extracellular Trap Release Is Regulated by Macrophage Migration-Inhibitory Factor” by Markryan Dwyer et al. had the highest burst strength (strength 6.74).

### 3.7 Keywords analysis of research hotspots

Using CiteSpace and VOSviewer for keyword co-occurrence, timeline, clustering, and burst analysis helps understand the research hotspots, frontiers, and trends in this field. According to VOSviewer statistics, there are 1250 keywords across 220 articles. If these keywords had similar means, they were merged. Through the analysis of keywords, we can understand the current situation of NETs in the field of fibrotic diseases.

**Figure 7:**
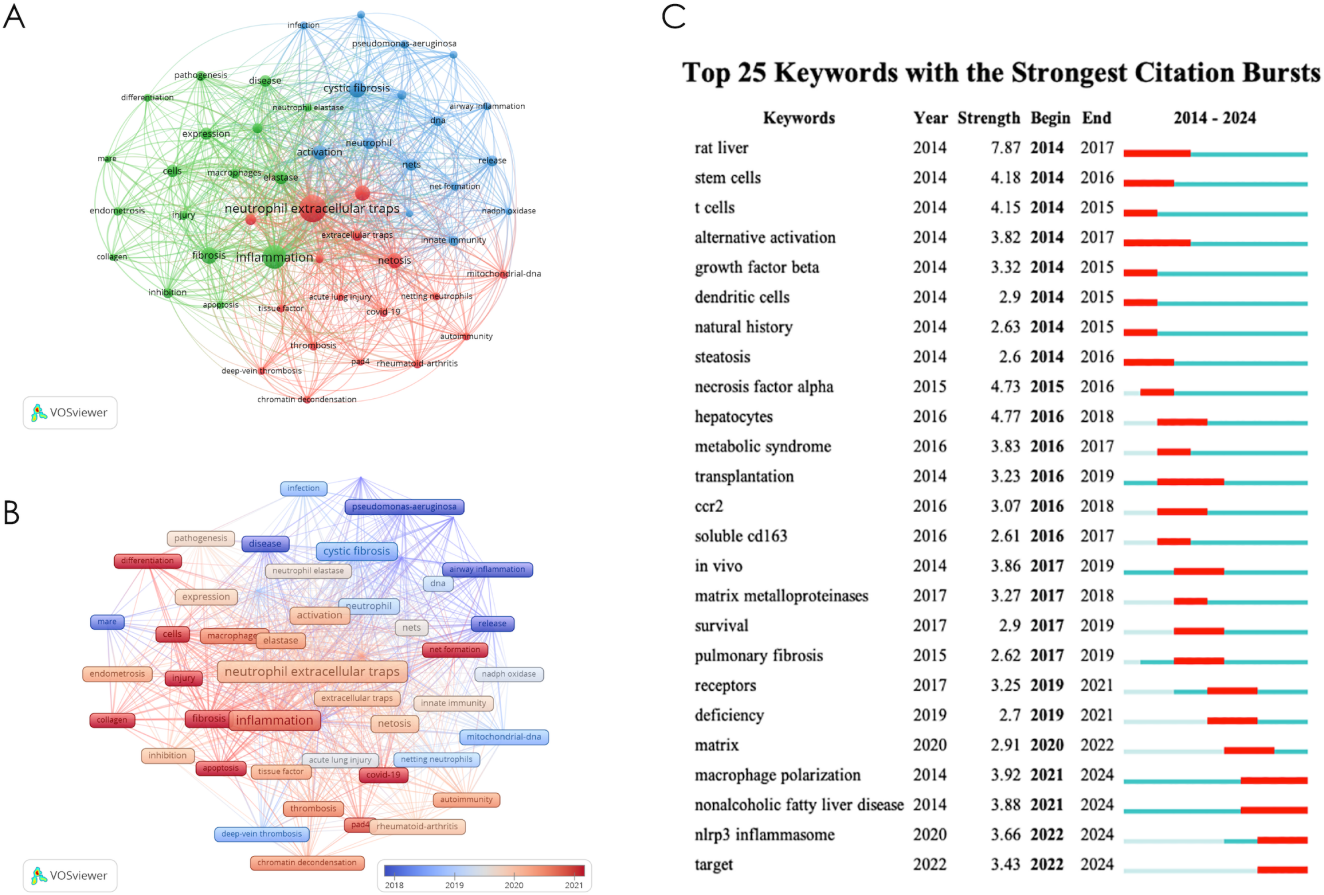
**(A)** keyword co-occurrence diagram; **(B)** Timeline diagrams of keywords; **(C)** Top 25 keywords with the strong citation bursts.

We employed CiteSpace and VOSviewer to analyze keyword co-occurrence in NETs-related fibrosis research (**Figure 7A**). The identified research hotspots can be broadly categorized into three main areas. The first group, represented in red, focuses on the pathological mechanisms of NETs in immune thrombosis, including thrombosis, deep vein thrombosis, contributing factors, COVID-19, and rheumatoid arthritis. The second group, denoted in green, emphasizes the immune response and fibrosis promoted by NETs, covering terms such as apoptosis, collagen, inflammation, and macrophages. The third group, shown in blue, highlights fibrotic diseases associated with NETs, including airway inflammation, cystic fibrosis, Pseudomonas aeruginosa, and lung disease. Furthermore, our co-occurrence analysis revealed keywords such as “chromatin decompaction,” “NET formation,” and “nuclear membrane rupture,” which are critical to the key steps of nuclear DNA extrusion, extracellular release, and NET formation. Although these terms do not form an independent cluster, they represent important findings that deepen our understanding of NETosis, which is also a prominent research focus within the NETs field.

**Figure 7B** shows the visualization of keyword time overlap. The earliest key is displayed in purple and blue; The latest keywords are displayed in orange and red. The results showed that the early research on NETs and fibrotic diseases mainly focused on airway inflammation, pseudomonas-aeruginosa, disease, release, etc. At this stage, the research mainly focused on proving the accumulation of NETs in tissues in cystic fibrosis, pulmonary fibrosis and other disease models. The latest research mainly focuses on net formation, apoptosis, collagen, injection, etc., suggesting that the recent research focuses on the mechanism that the accumulation of noose NETs aggravates or promotes the fibrosis process. The above analysis results are helpful for researchers to speculate on the future development direction of this field.

**Figure 7C** presents a keyword time overlap visualization. Keywords from earlier studies are depicted in purple and blue, while those from more recent research are shown in orange and red. The results indicate that early studies on NETs and fibrotic diseases predominantly focused on airway inflammation, Pseudomonas aeruginosa, and the pathogenesis and release of NETs. During this period, research primarily concentrated on the accumulation of NETs in tissue in disease models, such as cystic fibrosis and pulmonary fibrosis. In contrast, more recent studies have shifted toward topics like chromatin decompaction, NET formation, macrophages, and apoptosis, suggesting a focus on uncovering novel mechanisms of NETosis and its role in promoting fibrosis. These findings provide valuable insights for researchers in predicting future trends in this field.

**Figure 7C** shows the top 25 keywords of the strongest reference burst. It is worth noting that the citation outbreak of some keywords, such as pulmonary fibrosis (2020–2024), dam (2022-2024) and immunity (2022-2024), lasted until 2024, which means that related research fields are still widely concerned today.

## 4 Discussion

### 4.1 Global trends on NETs in fibrosis diseases

As far as we know, this is the first bibliometrics study on NETs and fibrotic diseases. Our results reveal significant findings, research interests and frontiers in this particular area. **Figure 1** shows the global growth trend of NETs publications in the field of fibrotic diseases from 2010 to 2024. In the last five years, the literature on NETs and fibrotic diseases has increased rapidly. The exponential growth of literature on neutrophil extracellular traps (NETs) and organ fibrosis since 2010 can be attributed to three interconnected factors: 1) seminal studies have established NETs as pivotal mediators of fibrotic progression across multiple organ systems, including the lung, liver, and kidney. Key discoveries have catalyzed interdisciplinary investigations into their pathophysiological roles; 2) cutting-edge methodologies have revolutionized NETs research. Innovations in single-cell sequencing, multiplex immunofluorescence imaging, and mouse models of fibrosis have made the spatiotemporal dynamics of NETs precise characterization of NETs; 3) strategic initiatives by major funding bodies have prioritized fibrotic disease mechanisms. Therefore, we assume that although the research on NETs and fibrotic diseases is still in its infancy, this field will continue to grow in the next few years.

According to the distribution of countries/regions (**Figure 2**), the United States is the countries with the largest proportion of published documents in this field. Correspondingly, Harvard University and University of Georgia in the United States are also in an important position in the institutional cooperation network. Despite the increasing volume of Chinese publications, the limited frequency of international collaborations in Chinese-led research may hinder its visibility and reduce editorial confidence, thereby restricting the ability of Chinese institutions to publish in high-impact international journals. Furthermore, many studies from China have primarily focused on validating established NET-fibrosis pathways in disease models, rather than introducing novel mechanisms, which may diminish their appeal to top-tier journals. Additionally, Chinese researchers often face language and presentation challenges as non-native English speakers, which can further impede the dissemination of their work. We argue that addressing these barriers will significantly enhance the global influence of Chinese institutions in this field.

Among the journals shown in **Table 2**, Frontiers in Immunology, International Journal of Molecular Sciences and Plos One may be the leading journals in the field of fibrotic diseases in NETs, especially the molecular, biological and genetic related sections. In addition, among the top ten authors, Rebordao Maria Rosa and her team have published the most papers and played a major role in this field.

### 4.2 Hotspots and emerging frontiers in NETs in fibrosis diseases

The reference of citation explosion (**Figure 6**) shows the widely cited literature in this field, shows the work that scientists are interested in at different stages, and may partially highlight the changes and trends of research in this field. Most of the early references on outbreak are related to the mechanism of NETs formation and release (16, 17). In 2010, Veronica Marcos et al. first reported that NETs are rich in the airway fluid of cystic fiber patients and mouse models (18). Although the manuscript was withdrawn one year after its publication, this article still attracted the attention of the industry in a short time and was cited 159 times. The role of NETs in the pathological process of cystic fibrosis has been continuously focused on in the subsequent citation outbreak literature (19–21). With the deepening of research, the effects of NETs-derived components such as Neutrophil elastase and Histones have been discovered (22). The review published by Samir Rahman et al. in Front Immunol in 2014 summarized the new understanding of NETs in cystic fibrosis (23). After that, the direction of citing outbreak literature gradually diversified, and diseases such as aging-related organ fibrosis (24), COVID-19-related pulmonary fibrosis (25), hepatic fibrosis and systemic sclerosis are gradually gaining attention, and research is progressively deepening to explore the pathological mechanism.

The strongest citation trends associated with specific keywords can serve as predictors for the future trajectory of network research in the field of fibrotic diseases. The results of the keyword co-occurrence analysis highlighted key terms such as “NETosis mechanism,” “immune response,” “fibrosis,” “tissue damage,” and “immune thrombosis.” In the subsequent sections, we will provide a detailed overview of the latest research findings related to these topics.

#### 4.2.1 The key mechanism of NETosis

Chromatin decondensation or dissolution is considered a prerequisite for NET formation. PAD4-mediated histone citrullination has been identified as a key driver of this process (8). PAD4 facilitates chromatin decondensation by disrupting the tight interaction of histone H1, which is involved in chromatin compaction (26). Recent studies have expanded the role of citrullination beyond histones, including its impact on proteins associated with nuclear and chromatin structures, such as LMNB1, LBR, VIM, and actin filament-related proteins. These findings add a layer of complexity to our understanding of PAD4’s role in NETosis (27). Additionally, histone acetylation contributes to chromatin decondensation by neutralizing positive charges, weakening chromatin’s overall structure and enabling the binding of various proteins that trigger transcriptional programs. These genome-wide transcriptional events are essential for chromatin depolymerization (28). Furthermore, neutrophil elastase (NE) has been recognized as a critical factor in chromatin depolymerization (29). NE translocates from cytoplasmic granules to the nucleus via yet-to-be-identified mechanisms, where it promotes chromatin decondensation by cleaving histones (30). Under the synergistic effect of the above mechanisms, the thick chromatin is decondensed and dissolved, making it possible for it to be released into the extracellular.

It is widely believed that the nuclear envelope represents the first physical barrier that must be breached for NETs release. The nuclear envelope consists of the outer and inner nuclear membranes and the nuclear lamina, which provides structural support. The nuclear lamina is composed of A-type and B-type lamins (31). Recent studies have reported new findings regarding lamin degradation and nuclear envelope rupture. During NETosis, PKCα, which is localized in the cytoplasm of resting neutrophils, translocates to the nucleus. Activated PKCα phosphorylates lamin B, facilitating nuclear envelope rupture. Pharmacological inhibition of PKCα has been shown to suppress NET formation *in vitro* (9). CDK4/6 has also been found to phosphorylate lamin A/C, contributing to nuclear envelope rupture. The use of CDK4/6 inhibitors (abemaciclib/LY2835219) effectively inhibits NET formation in human neutrophils (10). In addition to kinases, calpain-mediated proteolysis also plays a role in nuclear envelope rupture. Recent evidence suggests that Ca^2+^ influx leads to sustained elevation of cytosolic and nuclear Ca^2+^ levels, which activate calpain, causing degradation of the actin cytoskeleton and nuclear nesprin-1. The structural disruption of nesprin-1 leads to the disappearance of nuclear envelope morphology. Calpain inhibitors have been shown to suppress NET formation through this mechanism (32).

**Figure 8:**
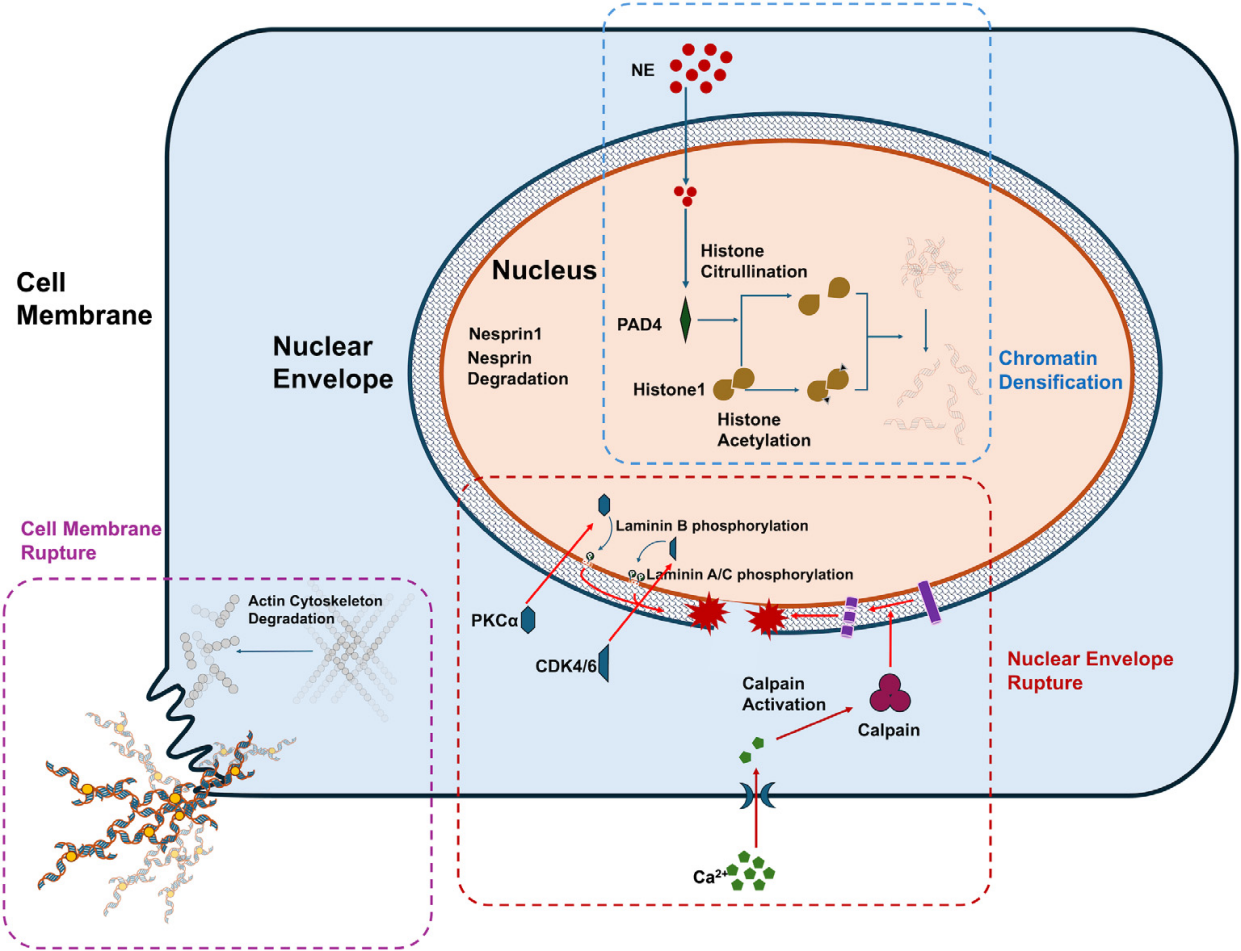
The key mechanism of NETosis.

The second physical barrier released by NETs is the plasma membrane. The cortical actin cytoskeleton beneath the plasma membrane plays a crucial role in maintaining cell membrane stability. The rapid disintegration of the cortical actin cytoskeleton is a key event in the extrusion of NETs during the later stages of NET formation. Following stimulation, neutrophils from both mice and humans exhibit rapid breakdown of the actin cytoskeleton, leading to the shedding of membrane microbubbles, disintegration and remodeling of microtubules and intermediate filaments, and blistering of the plasma membrane (11). Additionally, NET formation can directly induce plasma membrane rupture through pyroptosis-related inflammatory factors. Gasdermin E (GSDMD), a pore-forming protein implicated in apoptosis and plasma membrane rupture, translocates to the plasma membrane where it forms pores (33). Recent studies have highlighted the role of GSDMD in NET formation. During NET formation, GSDMD is hydrolyzed and activated, which enhances membrane permeability and facilitates NET extrusion (34). Pharmacological inhibition of GSDMD has been shown to suppress NET release, and interestingly, neutrophil elastase (NE) may also participate in GSDMD activation (35). These findings provide new insights into the mechanisms by which NE contributes to NET formation. The above NETosis mechanism is shown in **Figure 8**.

#### 4.2.2 NETs-immune cell crosstalk

The inflammatory response characterized by the infiltration of immune cells such as neutrophils, macrophages, and monocytes is considered a key culprit in the development of organ fibrosis. The intricate relationships among these cells have been a focal point of interest and challenge. NETs, as products of neutrophils, contain various pro-inflammatory mediators such as cytokines and chemokines, providing a pathway for crosstalk between neutrophils and other immune cells, including macrophages and monocytes, thereby exerting a broad impact on the immune mechanisms underlying fibrotic diseases.

Current understanding of the crosstalk between NETs and macrophages is the most advanced. On one hand, NETs are involved in the phenotypic transformation of macrophages. NETs were found to promote the conversion of macrophages to myofibroblast-like phenotypes via the TGF-β1/Smad3 signaling pathway in a renal fibrosis model (36). Myofibroblasts are the primary cell type responsible for collagen deposition during the fibrotic disease process and play a significant role in tissue repair and pathological fibrosis. In a model of myocardial infarction, free DNA from NETs can enhance the proliferation of Mer tyrosine kinase/Major Histocompatibility Complex II macrophages (Mertk-MHC-IIlo-int) through the Toll-like receptor 9 pathway (37). Mertk-MHC-IIlo-int is a pro-inflammatory macrophage type that promotes extracellular matrix degradation and phagocytosis of cellular debris. This finding suggests that NETs may play a beneficial role in ventricular remodeling by inducing specific immune responses in macrophages. The contradictory evidence may relate to the high plasticity and functional diversity of macrophages in chronic inflammation and tissue injury.

On the other hand, NETs facilitate macrophage infiltration and the subsequent release of pro-inflammatory cytokines from macrophages. In a heart failure with preserved ejection fraction (HFpEF) model, NETs were associated with macrophage infiltration and inflammatory responses; the breakdown of NETs by DNase 1 significantly reduced macrophage numbers in cardiac tissue and decreased IL-10 expression in macrophage (38). Additionally, NETs can elevate α-SMA levels in macrophage (39), a mechanism that plays an important role in renal fibrosis induced by hyperuricemia. In a mouse model of pulmonary fibrosis, NETs were found to antagonize the production of the anti-fibrotic cytokine IL-27 in macrophages, exacerbating tissue remodeling and fibrosis (40).

Recent findings also shed light on the crosstalk between NETs and monocytes. In a mouse model of MASH disease, NETs induced the production of pro-inflammatory factors such as IL-1β and TNF-α in monocytes, triggering the recruitment of monocyte-derived macrophages and the activation of senescent cells, with the NLRP3 signaling pathway potentially involved in this process (41).

#### 4.2.3 Fibrosis and tissue damage

Fibroblasts are key effector cells in the process of organ fibrosis, and NETs can influence their activation and proliferation through various mechanisms. NETs have been shown to target classical signaling pathways, such as TGF-β (42) and NLRP3 (41), promoting fibroblast activation, as evidenced by increased expression levels of biomarkers such as vimentin, α-SMA, and COL1A1. Additionally, NETs selectively impact the metabolic reprogramming of fibroblasts during the activation of hepatic stellate cells (HSCs) in liver fibrosis. HSCs co-cultured with NETs exhibit elevated oxygen consumption rates (OCR) and extracellular acidification rates (ECAR), indicating increased mitochondrial respiration and aerobic glycolysis. Further studies have demonstrated that the metabolic regulatory effects of NETs on HSCs are dependent on the arachidonic acid pathway, particularly involving cyclooxygenase-2 (COX-2) and Prostaglandin E2 (PGE2) (43).

Moreover, NETs can promote the epithelial-mesenchymal transition (EMT) in lung epithelial cells, inducing the expression of α-SMA, Snail, and Twist, while simultaneously decreasing E-cadherin expression, thereby facilitating lung fibrosis (44). The role of NETs in driving EMT in lung epithelial cells has also been demonstrated in critically ill patients with COVID-19. Studies have shown that lung tissues from critically ill COVID-19 patients exhibit high expression of both epithelial and mesenchymal markers. In an *in vitro* model, it was confirmed that the EMT expression pattern induced by SARS-CoV-2 correlates with NETosis (25). These findings suggest that NETs play a crucial role in the pathogenesis of COVID-19-associated pulmonary fibrosis.

The cytotoxic effects of NETs can also impact parenchymal cells, leading to a series of pathological changes. NETs can reduce the resistance of bronchial and airway epithelial cells, increasing the paracellular flux of macromolecules, which induces apoptosis in airway cells. Furthermore, exposure to NETs has been found to cleave E-cadherin protein (45), processes that collectively contribute to the disruption of epithelial barrier function. Emerging evidence suggests that neutrophil extracellular trap formation (NETosis) is implicated in the pathogenesis of advanced heart failure. Histopathological analyses have demonstrated significant NETosis deposition accompanied by substantial neutrophil infiltration within myocardial tissue from end-stage heart failure patient (46). Mechanistically, this neutrophil-derived inflammatory response may drive the pathological progression from compensated to decompensated cardiac hypertrophy through dual mechanisms: 1) Sustained release of pro-inflammatory mediators that potentiate myocardial fibrogenesis, and 2) Direct cytotoxic effects causing cardiomyocyte death and extracellular matrix remodeling (47).

Research on the components of NETs further elucidates its profibrotic and tissue-damaging effects. Studies indicate that NETs contain antimicrobial proteins and histones, which exhibit high biological toxicity and are involved in inflammatory responses, epithelial-mesenchymal transition (EMT), and epithelial injury. Several reviews have summarized the pathological processes of NET components in pulmonary fibrosis (48–50). Among these, NE has been the subject of considerable research. NE is implicated in neutrophil-mediated airway inflammation and can also stimulate excessive mucus secretion in models of cystic fibrosis (51), damaging the defensive functions of epithelial cells (52). Furthermore, NETs contain bioactive inflammatory cytokines such as IL-17, which promote fibroblast differentiation. Co-culturing lung fibroblasts with IL-17-rich NETs *in vitro* results in a fibrotic phenotype characterized by increased CCN2 expression, enhanced migration and healing abilities, and elevated collagen release (53).

#### 4.2.4 Immune thrombus

NETs have been found to play a significant role in promoting a hypercoagulable state and thrombosis in patients with fibrosis, with coagulation imbalance considered one of the important reasons for the progression of fibrotic diseases, often associated with severe clinical manifestations (54, 55). The pro-thrombotic effects of NETs are complex; on one hand, they serve as activators of thrombosis, while on the other hand, they act as scaffolds that influence the stability of thrombi. The mechanisms by which NETs stimulate thrombus formation may be related to the expression of tissue factor (TF) and IL-17A by NETs. Studies have shown that NETs extracted from the peripheral blood of patients with active systemic lupus erythematosus can express active TF and IL-17A, inducing thrombin production that activates the coagulation mechanism and promotes HSC activation (14). NETosis and the immunothrombosis induced by NETs play critical roles in COVID-19-related conditions such as pulmonary fibrosis and nonalcoholic steatohepatitis (NASH) (56). SARS-CoV-2 infection triggers complement activation accompanied by both neutrophil TF expression and NETs carrying active TF production, increasing its procoagulant activity (57). SARS-CoV-2 has been implicated in a range of lung diseases, including pulmonary nodules. Activated neutrophils have been detected in the lung tissues of patients with pulmonary nodules, accompanied by the formation of NETs (58). These findings suggest that NETs may play a role in the sequelae of COVID-19, however, further studies are required to substantiate this association.

Additionally, key components of NETs, such as free DNA, also exhibit pro-coagulant properties. Upon entering the plasma, free DNA binds to factor XII (FXII), triggering the production of thrombin. Furthermore, NETs serve as direct mediators of thrombosis (59). As complex extracellular structures, NETs provide a scaffold for the aggregation of platelets and fibrin. Pathological examination of alveolar tissues from patients who succumbed to severe COVID-19 revealed that NETs are important constituents of microvascular and macrovascular thrombi (60). When microvascular thrombosis occurs, it triggers a cascade of inflammatory responses, involving immune cells and fibroblasts. This amplifies the inflammatory response, further exacerbating ECM deposition, which may be one of the mechanisms by which NETs promote the formation of immune thrombus and contribute to fibrosis (61). These findings collectively suggest that NETs represent reliable and promising therapeutic targets for COVID-19-related lung diseases. Current research on therapeutic strategies has primarily focused on the direct degradation of NETs and the inhibition of key pathways involved in NETosis, with DNase I and PAD4 inhibitors serving as key examples. The pharmacological effects of these strategies are mainly centered on anti-inflammatory and antithrombotic therapies. Among these, recombinant human DNase (rhDNase) is an FDA-approved drug that promotes the degradation of NETs by catalyzing the hydrolysis of extracellular DNA. Administration of rhDNase has been shown to reduce the inflammatory response, decrease platelet activation, and improve local blood flow in mouse models of acute respiratory distress syndrome (ARDS) (62).

## 5 Summary and outlook

This study combines bibliometric analysis with a review of the research hotspots related to the pathological mechanisms by which NETs participate in fibrotic diseases, offering certain advantages over previous studies that relied solely on bibliometric analysis or narrative reviews. To our knowledge, this is the first bibliometric study focusing on the relationship between NETs and fibrotic diseases. It is important to note that the bibliometric analysis is limited to literature retrieved from the WoS, which may result in an incomplete collection of relevant studies. Consequently, the discussions presented in this paper have certain limitations.

Although our findings indicate a rapid increase in the literature concerning NETs in the context of fibrosis, several significant challenges and unresolved issues remain. First, the intricate mechanisms underlying NETosis are still not fully elucidated. For instance, the initial triggers of NETosis remain unclear, as does the sequence of events, such as whether chromatin condensation occurs prior to or simultaneously with the rupture of the nuclear membrane. Addressing these questions will provide a more comprehensive understanding of this complex process and potentially inform the development of targeted therapeutic strategies aimed at NETs to prevent fibrotic diseases. Second, the crosstalk between neural networks and immune cells represents a highly complex process with numerous unresolved questions. Specifically, it remains uncertain whether NETs interact with lymphocytes, and the precise mechanisms by which immune cell crosstalk promotes fibrosis are yet to be determined. Furthermore, the role of NET components in the promotion of fibrosis is still unclear, with most studies focusing on pulmonary fibrosis models. The involvement of enzymes such as neutrophil elastase (NE) and myeloperoxidase (MPO) in myocardial and liver fibrosis remains poorly understood. Future research should aim to clarify the specific mechanisms by which NETs contribute to fibrotic diseases, including their interactions with a broader spectrum of immune cells, the pathways through which NETs activate fibroblasts, and the potential cytotoxic effects of NETs themselves. Based on our findings, these areas represent critical directions for future investigation.

## Data Availability

The original contributions presented in the study are included in the article/supplementary material, further inquiries can be directed to the corresponding author/s.
